# Ocular following Eye Movements in Marmosets Follow Complex Motion Trajectories

**DOI:** 10.1523/ENEURO.0072-23.2023

**Published:** 2023-06-09

**Authors:** Jagruti J. Pattadkal, Carrie Barr, Nicholas J. Priebe

**Affiliations:** 1 Center for Learning and Memory, The University of Texas at Austin, Austin, Texas 78712; 2 Department of Neuroscience, The University of Texas at Austin, Austin, Texas 78712

**Keywords:** intersection of constraints, marmoset, motion, ocular following, plaids

## Abstract

Ocular following eye movements help stabilize images on the retina and offer a window to study motion interpretation by visual circuits. We use these ocular following eye movements to study motion integration behavior in the marmosets. We characterize ocular following responses in the marmosets using different moving stimuli such as dot patterns, gratings, and plaids. Marmosets track motion along different directions and exhibit spatial frequency and speed sensitivity, which closely matches the sensitivity reported in neurons from their motion-selective area MT. Marmosets are also able to track the integrated motion of plaids, with tracking direction consistent with an intersection of constraints model of motion integration. Marmoset ocular following responses are similar to responses in macaques and humans with certain species-specific differences in peak sensitivities. Such motion-sensitive eye movement behavior in combination with direct access to cortical circuitry makes the marmoset model well suited to study the neural basis of motion integration.

## Significance Statement

Ocular following is a reflexive eye-tracking behavior in response to large visual field motion. It reflects the properties of underlying motion-sensing circuits. One of the primary motion-sensing areas in primates is the area MT. In the primate species of marmosets, this and other cortical areas are easily accessible due to their lissencephalic brain. We demonstrate ocular following behavior in the marmosets for simple and complex motion trajectories and describe its characteristics. We then use ocular following to distinguish between different motion integration models. Our results show the utility of ocular following to study the neural basis for motion sensing in marmosets.

## Introduction

A critical function of sensory processing is to integrate distinct information from our environment to generate a global representation to guide our behavior. As information flows through the visual system, transformations occur that integrate multiple signals to constrain the interpretation of our visual environment. An example of this process occurs in the visual system when inferring the motion of complex objects. This inference requires the integration of multiple visual motion signals, each providing a constraint on the interpretation of the global object motion, seen for instance in our interpretation of overlapping moving grating stimuli—a plaid that appears to move in the direction distinct from either of the grating stimuli. The emergent representation of this pattern motion provides us with a model system to examine the neural circuitry that underlies signal integration in cortex.

Motion signal integration can occur following different principles such as vector averaging (VA) or intersection of constraints (IOC; see below). The motion of the individual grating components in a plaid stimulus is ambiguous and could correspond to a set of different motion interpretations. This ambiguity is known as the “aperture problem.” For a single grating, the interpretations of its motion lie along a line in the velocity space, and we generally perceive the motion corresponding to the slowest velocity. Overlapping gratings moving in different directions constrain our interpretation of motion: in this case, plaid motion can be computed based on the VA of the motion direction of the components (Yo and Wilson, 1992) or by considering a more complex model in which each motion component contributes a constraint line, and the intersection of constraints lines from multiple components yields our percept (IOC; Adelson and Movshon, 1982). For cases in which the two gratings are moving at the same speed (“type I plaids”), the VA and IOC models yield the same direction. Plaid stimulus where two motion components move at different speeds (“type II plaids”), can yield IOC predictions that differ from VA predictions by lying outside the two motion components. The VA predictions always lie between the motion components. Humans generally perceive motion in the IOC direction (Ferrera and Wilson, 1990), though this percept depends on the contrast of the motion components and the duration of stimulus presentation (Yo and Wilson, 1992).

We can infer the interpreted motion by using the eye movements evoked in response to the moving patterns. Brief presentations of large moving stimuli evoke reflexive ocular following eye movements that aid in stabilizing the visual world on the retina (Kawano and Miles, 1986; Miles et al., 1986; Miles and Kawano, 1986; Miles, 1998). Ocular following in macaques depends on activity in motion-selective neurons in the dorsal visual pathway including areas like middle temporal area (MT) and medial superior temporal area (MST; Kawano et al., 1994; Takemura et al., 2007). The direction of human ocular following eye movements in response to the moving plaids is biased toward the IOC direction, particularly at long latencies (Masson and Castet, 2002; Quaia et al., 2016). The eye movement behavior can be used to gain insights into the motion integration computation performed by the motion pathway. We examine ocular following responses (OFRs) of marmosets. These animals are particularly amenable to studying motion computations because of their lissencephalic brain. The motion-selective areas like MT and MST all lie on the brain surface and are easy to access for neural recordings (Pattadkal et al., 2022), unlike in the macaque brain. We demonstrate that marmosets are a good model for studying ocular following behavior and describe the ocular following response properties in these animals using different stimuli such as a field of moving dots, gratings, as well as type II plaids. Marmoset ocular following responses match those found in humans, though the spatial parameters eliciting peak responses differ in ways that reflect the spatial resolution of the marmoset visual system.

**Table 1:** Comparison of the ocular following movements in human, macaque, and marmoset

	Human	Macaque	Marmoset
Latency for sine-wave gratings moving at 20–25°/s (ms)	72–82	55–60	38–53
Peak spatial frequency (cpd)	0.1	0.05–0.2	0.3
Peak speed (°/s)	40	200	25–50

Values are based on data from the following studies: Gellman et al. (1990) for Human; Miles et al. (1986) and Miura et al. (2014) for Macaque; and the present study for Marmoset.

## Materials and Methods

Eye movements were measured from four marmoset subjects (three males, one female) who were trained to fixate small spots or faces. Before training, a headpost was affixed to the skull in a sterile procedure performed in isoflurane anesthesia. The titanium headpost was affixed to the skull using Metabond (Parkell). Once the marmosets recovered from the headpost procedure, they were trained to receive juice reward for fixating small spots or faces (Mitchell et al., 2015). Subjects were maintained on food control to provide motivation in behavioral tasks with their weight ranging from 5% to 10% of baseline weight calculated as the average weight during a 4 week period each year when the animal is provided with food and water *ad libitum* and no behavioral training occurs. All procedures conformed to National Institutes of Health guidelines and the guidelines of the University of Texas at Austin Institutional Animal Care and Use Committee.

### Experimental procedures

Eye movements were measured using the Eyelink 1000 Plus system for infrared eye tracking. The data were collected from the right eye in pupil-CR mode at 1 kHz. Stimuli were displayed on a gray background screen (mean luminance = 31.5 cd/m^2^). The task control and data collection were performed using Maestro software (https://sites.google.com/a/srscicomp.com/maestro/). Animals were initially required to fixate on a target 5° from the central fixation for 500–800 ms, and then the target was shifted to the center of the screen. Animal was required to fixate on a center target for 250–300 ms with a 220 ms grace period. Fixation is not required in the grace period, but target is available and animals can begin fixation at any time during the grace period. Once the animal made this saccade to the central target, the target was extinguished, and a dot field, grating, or plaid stimulus was presented and moved for 200–400 ms. This randomized presentation duration was the same for all stimuli. No requirement for eye movement was made for the motion stimulus, and if the animal performed the initial saccadic eye movement from the distal to central target, they received a juice reward at the end of the trial, after stimulus was turned off. The intertrial interval varied between 1 and 2 s on different days.

All stimuli were presented on a FlexScan T761 50 cm (19 inch) Class Color Display cathode ray tube monitor with a refresh rate of 85 Hz. This monitor was placed 50 cm away from the marmoset, and field of view was 35° by 28°.

### Preprocessing of eye movements

We observed that baseline eye positions following fixation to a second target tended to drift. This may be similar to the glissades following saccades reported in previous studies (Kawano and Miles, 1986). Averaging the responses to the saccades only, without a motion stimulus (blank), can be used to cancel these drifts. However, we observed that this drift was not common across all trials, even when the saccade direction was the same. The amount of drift in the initial period, before the moving target can affect eye movement, is not related to the motion response of the eye movement later in the trial. But we have chosen to exclude trials that show this drift, by enforcing a threshold criterion for position deviation. We smoothed horizontal and vertical eye positions using a median filter with an order of 30. The maximum deviation is calculated from these smoothed positions during an interval starting 55 ms before motion onset to 25 ms after motion onset. Any trial with position deviation exceeding 0.2° in either vertical or horizontal position was excluded from our data. For animals A, B, C, and D, overall 48%, 24%, 47%, and 52% of the trials were included, respectively. In addition, trials with any saccade onset in a period of 150 ms after motion onset were also excluded from our analysis.

#### Eye velocity calculation

To compute the eye velocity, an eye position trace collected at 1 kHz was median filtered with an order of 25. The trace was then differentiated at 50 and 25 Hz, and the mean was used as an eye velocity estimate (Mitchell et al., 2015).

#### Latency calculation

To estimate the latency of ocular following responses, we first obtained an average eye velocity trace. This average was calculated across trials, sampled with replacement. The total number of trials averaged equals the number of trials in the dataset for that condition. The average eye velocity $e_{v}$ is then fitted with a threshold linear function of the following form:

$$e_{v}=g\left\lfloor (t-l)\right\rfloor +c,$$

where g is a gain factor, t is time of the trace being 0 at motion onset, l is the latency of the velocity trace, and c is the offset. We bootstrap this latency measurement by repeating the process 1000 times and use the median of the generated distribution as the mean latency for that condition. Latencies used for each test were calculated either for the conditions evoking strongest responses or averaged across different conditions when the responses had similar magnitude.

#### Open loop interval and response amplitude calculation

We have restricted our ocular following response amplitude measurements to the open loop period from motion onset. Open loop interval was estimated to end at twice the latency estimate (Masson and Castet, 2002). The amplitude of ocular following response was calculated by subtracting the average velocity before the start of the response (latency) from the average velocity at the end of the open loop interval. The average velocities were computed in a 15 ms period before the latency and from 15 ms period before the end of the open loop interval defined as twice the latency.

#### Sinusoidal fit for dependence of OFR on motion direction

The horizontal and vertical eye velocities were fitted with sinusoidal function, as follows:

$$e_{v}=\alpha \;\sin\left(\theta_{\text{stim}}-\theta_{\text{pref}}\right)\;+\;c,$$

where $e_{v}$ is the average eye velocity, α is the amplitude, $\theta_{\text{stim}}$ is the stimulus direction, $\theta_{\text{pref}}$ is the preferred stimulus direction, and c is the offset term.

Circular correlation between ocular following angle $\left(\theta_{\text{em}}\right)$ and stimulus direction $\left(\theta_{\text{stim}}\right)$ was computed using the following formula (Batschelet, 1981):

$$r=\frac{1}{n}\sqrt{\left[{\left(\sum^{}\cos\left(\theta_{\text{em}}-\theta_{\text{stim}}\right)\right)}^{2}+{\left(\sum^{}\sin\left(\theta_{\text{em}}-\theta_{\text{stim}}\right)\right)}^{2}\right]},$$

where *n* is the number of conditions.

#### Gaussian fit for dependence on speed or spatial frequency

The OFR amplitude dependence on speed or spatial frequency (SF) was fit with a Gaussian function, as follows:

$$e_{v}=\alpha \;e^{\frac{-{\left({\text{log}}_{2}(x)-l{\text{og}}_{2}(x_{\text{pref}})\right)}^{2}}{2\sigma^{2}}}\;+\;c,$$

where $e_{v}$ is the average eye velocity, α is the gain factor, x is the stimulus speed or spatial frequency, $x_{\text{pref}}$ is the preferred stimulus speed or spatial frequency, σ is the SD of the Gaussian, and c is the offset term.

### Data availability

All data included in the figures are deposited on the figshare website link: https://doi.org/10.6084/m9.figshare.22695988.v1.

## Results

We aim to characterize OFRs and determine how they follow complex motion trajectories in the marmosets. OFRs have been characterized in a number of mammals, including rodents, macaques, and humans, but the visual parameters required to evoke these smooth eye movements differ across species. Because these eye movements have not previously been characterized in marmosets, we initially describe the visual parameters that elicit OFRs.

### Ocular following responses to simple motion stimuli

**Figure 1.**
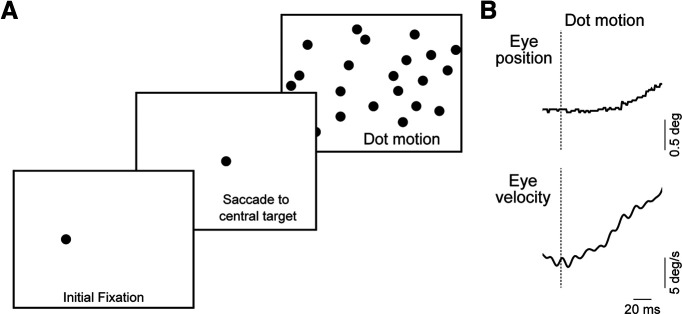
Ocular following task and example eye movement. ***A***, Schematic of the ocular following task. Trials began with an initial fixation target, 5° away from the center of the screen. Animals were then required to make a saccade to a central fixation target. Following this fixation, motion stimulus turned on, which was a field of dots, a grating, or a plaid. ***B***, Example ocular following response in a marmoset for a dot field motion. Top panel shows the eye position trace, and bottom panel shows the eye velocity trace. Dashed vertical line indicates the start of motion stimulus. deg, Degree.

To evaluate the visual parameters that evoke OFRs we used a saccade-initiated paradigm in which animals are required to make a saccadic eye movement from a 5° eccentric target to a central target, after which a motion pattern was presented (Fig. 1*A*). We used this saccade-initiated paradigm because it reduced the saccade instances within few hundred milliseconds after motion onset and because OFR is known to exhibit postsaccadic enhancement (Kawano and Miles, 1986). This is the interval used for analyzing ocular following responses, and trials with saccades during this interval were discarded (see Materials and Methods).

We initially presented moving patterns that were composed of randomly placed dots that varied in size from 0.6° to 1.6°. In response to rightward motion of the dot pattern at 25°/s, clear, smooth movements are evoked following the saccade to the central target (Fig. 1*B*). Smooth eye movements were initiated rapidly after the visual pattern movement (median latency: animal A, 57 ms; animal B, 63 ms). To quantify the mean OFRs, we measured the mean evoked eye velocity in a 15 ms period at the end of the open-loop interval and subtracted velocity before the estimated latency from it (see Materials and Methods). For rightward motion, the mean ± SEM OFR was 6.5 ± 0.2°/s in animal A and 4.5 ± 1.1°/s in animal B. OFR gain was comparable for both animals (mean gain: animal A, 0.26 ± 0.009; animal B, 0.18 ± 0.05).

**Figure 2.**
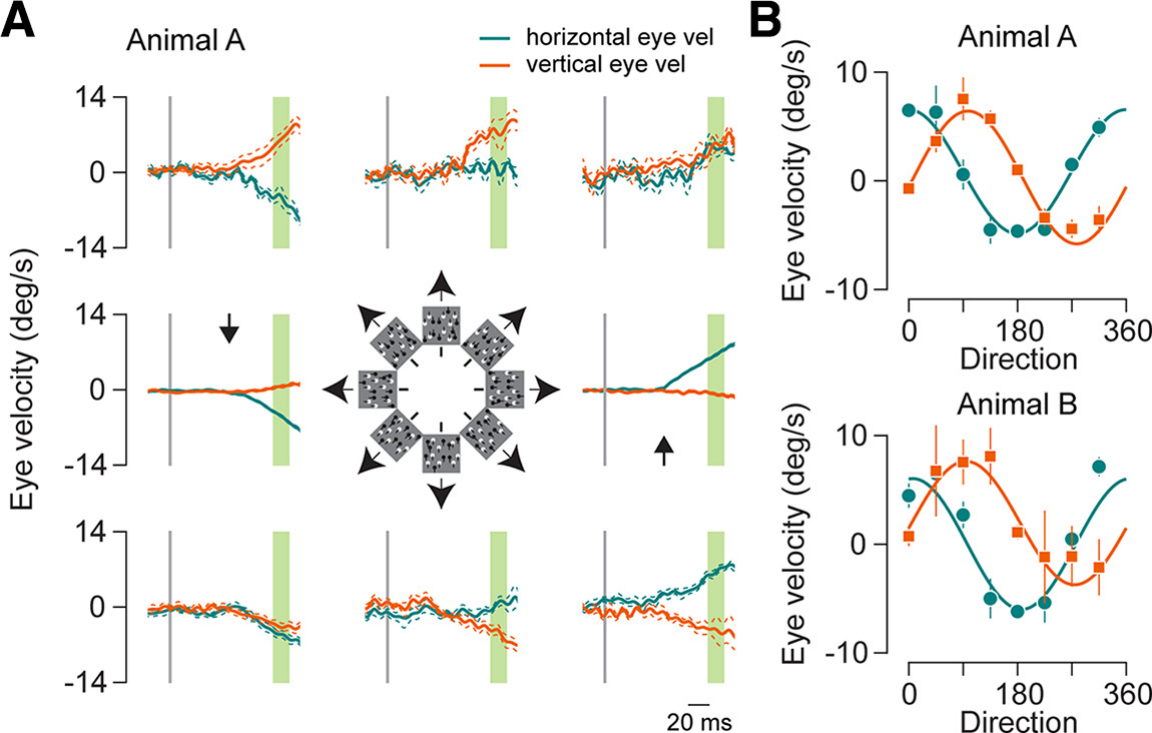
OFR to different directions. ***A***, Mean eye velocities for 8 different dot field directions for animal A. Panels are arranged in counterclockwise order with the right center panel corresponding to rightward or 0° motion. The cartoon in the center indicates the stimulus motion order. Thick traces in orange and green indicate the vertical and horizontal mean eye velocities. Thin traces indicate the SEM around the velocity. Vertical gray line shows the start of motion stimulus. Green highlighted region is used for calculating the amplitude of OFR. Latency was calculated as the mean of the rightward and leftward motion conditions and is shown by the black arrows. ***B***, OFR amplitude for horizontal and vertical eye velocity for different stimulus motion directions. Top panel is for OFR in animal A, and the bottom panel is for OFR in animal B. Orange points indicate vertical eye velocity, and green indicates horizontal eye velocity. Responses are fitted with sinusoidal function (see Materials and Methods). Error bars indicate 1 SEM. vel, Velocity; deg, degree.

Changing the direction of dot motion systematically shifted the horizontal and vertical eye velocity components (Fig. 2*A*), and no clear biases were observed in either marmoset. The mean eye-tracking direction of the ocular following responses closely matched the presented stimulus motion direction for both animals (Fig. 2*B*; circular correlation: animal A, 0.98; animal B, 0.94; trials: animal A: average number of trials per condition, 30.4; range, 5–102; animal B: average number of trials per condition, 12.1; range, 3–27).

**Figure 3.**
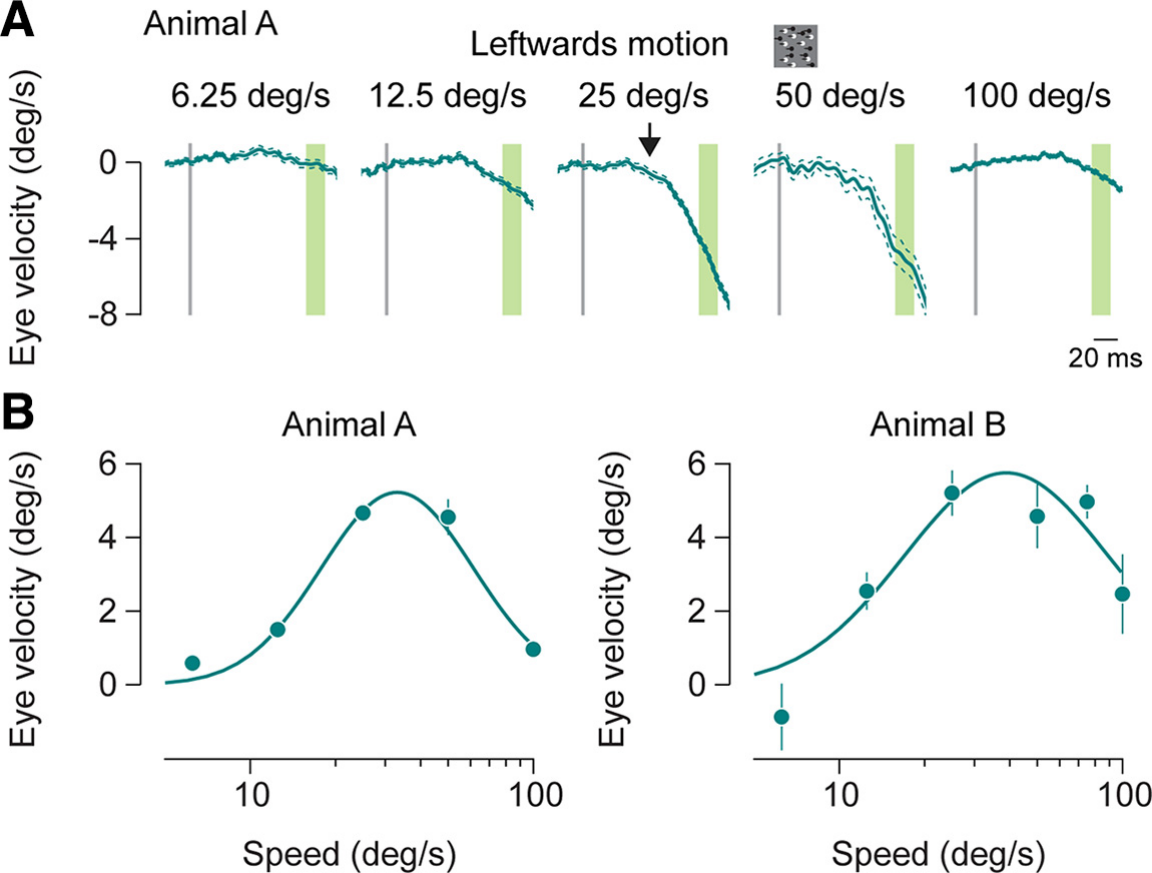
OFR to different speeds. ***A***, Mean horizontal eye velocity for dot fields moving leftward at different speeds. Speed is indicated at the top of each panel. Thick line indicates the mean eye velocity, and thin lines indicate the SEM. Gray vertical line shows the start of the motion stimulus. Latency is calculated for the 25°/s stimulus, shown by the black arrow. OFR amplitude is average from the period highlighted in green. ***B***, Mean OFR amplitude as a function of stimulus speed. Left panel shows responses from animal A, and right panel shows responses from animal B. Error bars indicate 1 SEM. Amplitudes are fit with a log Gaussian function. deg, Degree.

Having established that marmosets can exhibit ocular following eye movements in response to large field motion, we then examined the range of dot speeds over which OFRs are generated. Using the dot stimulus moving in either the right or left direction, we systematically adjusted the speed of the stimuli. The initial eye velocity of the OFRs increased with dot speed, peaking at ∼25–50°/s. Speed greater than that was not as effective in driving ocular following responses (Fig. 3*A*). The OFR amplitude dependence on speed of motion could be fit with a log Gaussian function (see Materials and Methods), with comparable mean and SD values for the two animals (Fig. 3*B*; rightward motion: animal A: fit mean, 29.2°/s; SD, 0.83 log units; animal B: fit mean, 31.2°/s; SD, 1 log unit; leftward motion: animal A: fit mean, 33.1°/s; SD, 0.79 log units; animal B: fit mean, 38.9°/s; SD, 0.92 log units; trials: animal A: average number of trials per condition, 73.8; range, 19–118; animal B: average number of trials per condition, 9.3; range, 3–19).

### Ocular following responses to single grating motion

**Figure 4.**
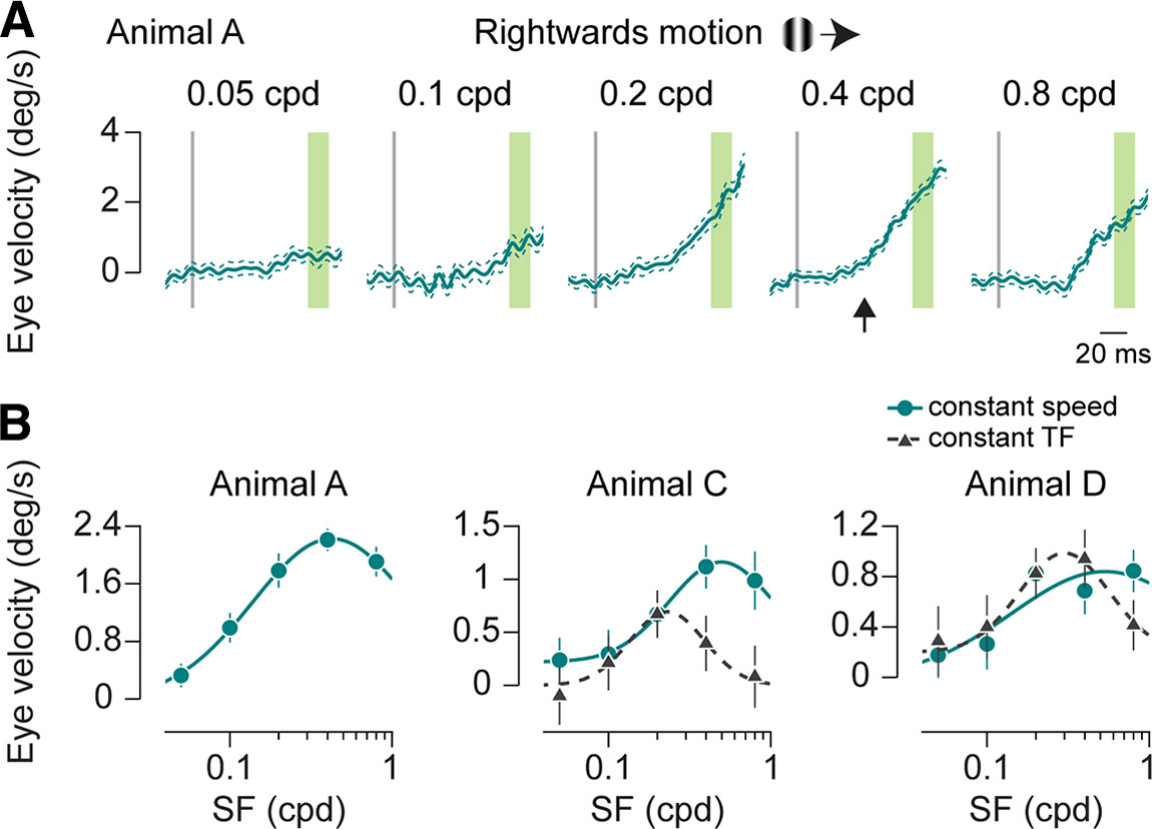
OFR to different spatial frequencies. ***A***, Mean horizontal eye velocity for single grating of different spatial frequencies moving rightward at 25°/s. Spatial frequency is indicated at the top of each panel. Thick line indicates the mean eye velocity, and thin lines indicate the SEM. Gray vertical line shows the start of the motion stimulus. Latency is calculated for the 0.4 cpd stimulus, shown by the black arrow. OFR amplitude is the average from the period highlighted in green. ***B***, Mean OFR amplitude as a function of stimulus spatial frequency. Each panel is data from a different animal, indicated in the heading. For animals C and D, data were collected using constant speed as well as constant temporal frequency conditions. Teal circles and continuous lines indicate data and fits for constant speed conditions; gray triangles and dashed lines indicate data and fit for constant temporal frequency conditions. Error bars indicate 1 SEM. Amplitudes are fit with a log Gaussian function. deg, Degree; TF, temporal frequency.

Our goal is to examine ocular following responses to combinations of moving gratings. To ascertain that we are using grating parameters that can evoke robust OFRs, we first measured the spatial frequency sensitivity of marmoset OFRs by presenting gratings moving at 25°/s at different spatial frequencies. We observe a clear dependence of OFR amplitudes on the spatial frequency of the gratings in both animals. The responses peaked at ∼0.2–0.5 cycles/° (cpd) and declined for both lower and higher spatial frequencies (Fig. 4). The responses were fitted with a log Gaussian function and had comparable mean and SD values for different motion directions and across animals (rightward motion: animal A: fit mean, 0.4 cpd; SD, 1.07 log units; animal C: fit mean, 0.5 cpd; SD, 0.87 log units; animal D: fit mean, 0.5 cpd; SD, 1.16 log units; leftward motion: animal A: fit mean, 0.4 cpd; SD, 0.94 log units; animal C: fit mean, 0.4 cpd; SD, 0.72 log units; animal D: fit mean, 0.4 cpd; SD, 0.82 log units). Another approach to measuring spatial frequency sensitivity is by presenting stimuli with different spatial frequencies at a constant temporal frequency, instead of constant speed. We measured spatial frequency sensitivity in this manner as well for two of the animals. In this case, gratings were presented at different spatial frequencies with varying speeds to maintain temporal frequency constant at 5 Hz. We find that the preferred spatial frequency is lower with the constant temporal frequency conditions, compared with constant speed conditions (rightward motion: animal C: fit mean, 0.2 cpd; SD, 0.74 log units; animal D: fit mean, 0.3 cpd; SD, 0.81 log units). This suggests that ocular following responses show separable sensitivity to stimulus spatial and temporal frequencies, rather than being speed tuned, consistent with previous results from macaque ocular following responses (Miura et al., 2014) and humans (Sheliga et al., 2016; trials: animal A: average number of trials per condition, 108.5; range, 90–148; animal C: average number of trials per condition, 56.2; range, 32–77; animal D: average number of trials per condition, 82.4; range, 46–115). Based on these responses, we have chosen the spatial frequency of 0.4 cpd for the experiments with the plaid stimuli described next.

### Ocular following responses to plaid motion

Both the dot and grating motion stimuli are composed of strong motion signals consistent with motion in a single direction. Our question, however, revolves around how multiple motion signals are integrated to generate ocular following responses. One approach to assess this is to present two overlapping gratings with distinct orientation and to measure how the motion of these grating components is integrated to elicit a behavior such as the OFR (Masson and Castet, 2002). As described earlier, multiple models exist to explain motion integration. Two of the models are vector sum (VS) and VA, in which the individual motion components are either summed or averaged to give the global motion interpretation. A third model is the IOC model in which each motion component provides a set of constraints to the global motion and the overall motion satisfies all constraints. We use a type II plaid stimulus to test for motion integration in marmosets because it distinguishes between IOC and VA models. This stimulus is formed by superimposing two gratings moving at different speeds along nearby directions. The IOC motion direction in this case falls outside of the two component motion directions (unlike type I plaids). If ocular following eye movement tracks this direction, it offers direct evidence of the IOC model of motion integration.

We used a variant of type II plaids, called unikinetic plaids, in which one grating component was stationary, oriented diagonally at 45°, and the other moved at 25°/s along the vertical direction, either upward or downward. The vector average and vector sum predictions are that motion signals are in the direction of the moving grating component as the second grating is static, whereas the IOC model predicts a computation of motion along an axis of orientation of the stationary grating. Presenting these type II plaids and examining eye movement along the horizontal axis (Masson and Castet, 2002), we cannot only test for the existence of motion integration but also distinguish between the models used for motion integration computation guiding eye movements. If IOC computations are used to generate OFRs, there should be a smooth eye movement in the horizontal direction, whereas if a VA or VS computation is used, there should be only vertical smooth eye movements.

**Figure 5.**
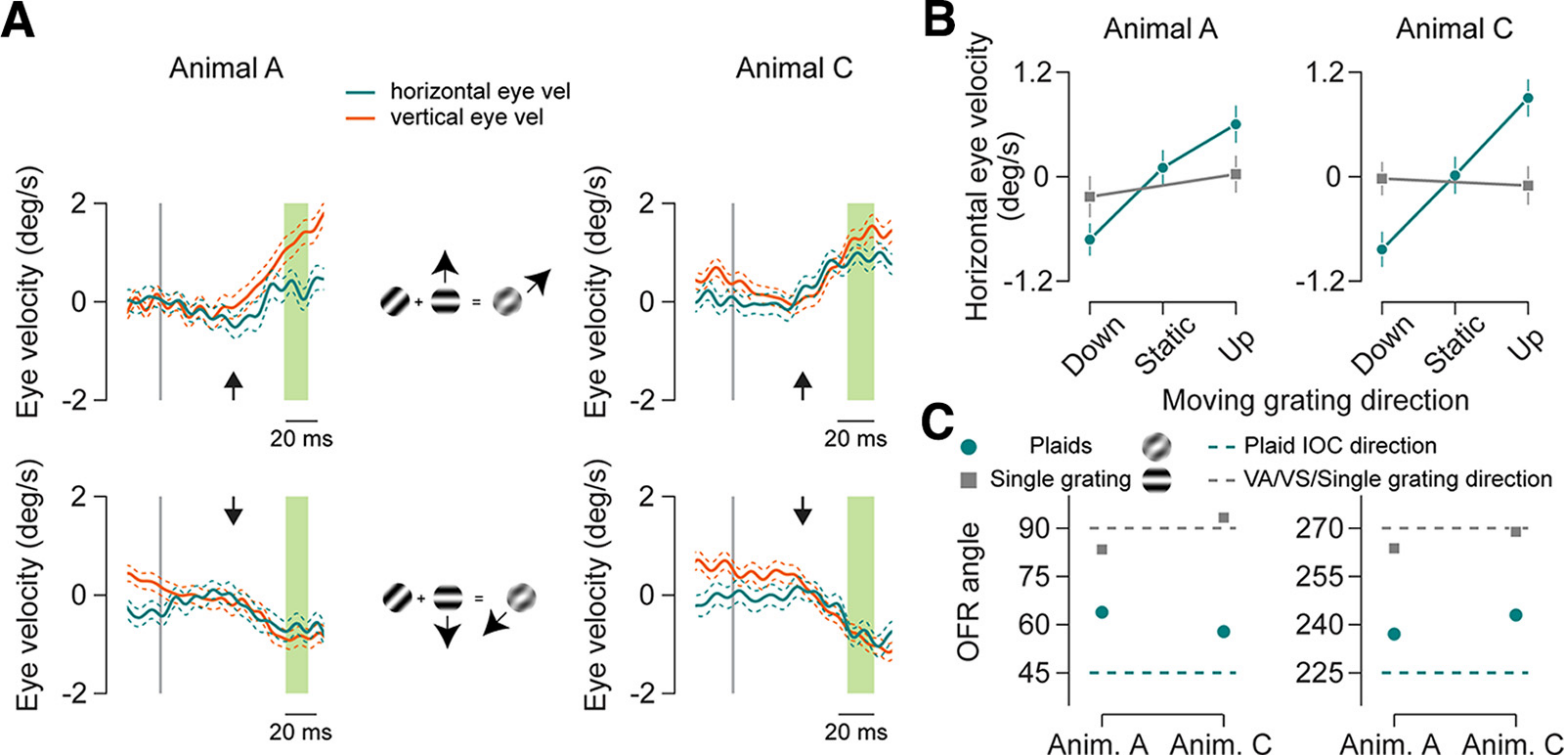
OFR to unikinetic plaids. ***A***, Mean eye velocity traces in response to unikinetic plaids motion. Top row shows responses for plaid composed of static diagonal grating and upward-moving grating. Bottom row shows responses for static diagonal and downward-moving grating. First column is for animal A, middle column shows the schematic for plaid composition, and third column is for animal C. Thick lines indicate median eye velocities, and thin dashed lines indicate SEM. Orange lines represent vertical eye velocity, and green lines indicate horizontal eye velocity. Vertical gray lines indicate the start of motion. Eye velocity is averaged from the green highlighted section. Latency of responses is indicated by the black arrows. ***B***, Mean horizontal eye velocity for plaids along different directions. Left panel is for animal A, and right panel is for animal C. Green circles indicate velocity in response to plaids. Gray boxes indicate horizontal velocity for single gratings moving upward or downward. Error bars are 1 SEM. ***C***, Ocular following angle for plaids and grating stimuli. Left panel is for plaid or grating with moving component along upward direction and right panel is for plaid or grating with moving component along downward direction. Green circles are OFR angles for plaid motion, and gray boxes are OFR angles for single grating motion. The direction of the moving grating component is indicated by the dashed gray line, and the IOC direction for plaid motion is shown by the green line. Error bars (smaller than points) indicate 1 angular SD. Anim., Animal; vel, velocity.

We measured smooth eye movements in response to unikinetic plaids and found a component of eye velocity trajectories consistently in the horizontal direction for both marmosets (Fig. 5*A*), consistent with the IOC model. To estimate whether the horizontal eye velocity observed in response to unikinetic plaids was different from noise, we compared its magnitude to the magnitude of horizontal eye velocity observed in response to single gratings moving in the vertical direction. The horizontal component in response to single grating motion along the vertical direction gives us an estimate of the noise in the horizontal eye velocity magnitude. We find that the horizontal eye velocity in response to plaid motion was higher than the noise estimate for both upward and downward grating motion in both animals (Fig. 5*B*; upward motion *t* test: animal A, *p* = 0.03; animal C, *p* = 0.0007; downward motion *t* test: animal A, *p* = 0.05; animal C, *p* = 0.002; trials: animal A: average number of trials per condition, 70.7; range, 50–83; animal C: average number of trials per condition, 71.3; range, 63–78), indicating a significant response along the horizontal direction consistent with the IOC model. The results remain unchanged when using the entire open loop period to calculate the OFR amplitude (upward motion *t* test: animal A, *p* = 0.06; animal C, *p* = 0.0008; downward motion *t* test; animal A, *p* = 0.02; animal C, *p* = 0.03).

At the end of the open loop interval, the mean direction of the smooth eye movements was along the diagonal for both animals (Fig. 5*C*; plaid with upward moving grating: circular mean ± angular SD tracking angle: animal A, 63.9 ± 1°; animal C, 57.8 ± 1°; plaid with downward moving grating: circular mean ± angular SD tracking angle: animal A, 237.1 ± 1.1°; animal C, 243 ± 1°). Note however, that while these horizontal eye movements are consistent with a contribution of the IOC model to the integration of motion trajectories, these angles systematically underestimate the expected angle, given the IOC model (45° for plaid with upward moving grating and 225° for plaid with downward moving grating).

## Discussion

The New World primate species of marmosets offers exciting advantages as a system to study the link between sensory representations and action: these primates exhibit complex cognitive skills, have lissencephalic brains providing easy access to the neocortex, and have well characterized sensory and motor areas. We have identified the characteristics of the visual motion signals required to elicit ocular following responses in marmosets, which may be used to study the link between neural responses and behavior. In addition, we have demonstrated that marmosets resolve complex motion patterns consistent with an intersection of constraints model, as in other primates. Overall, marmoset ocular following responses are similar to those of humans and macaques, with species-specific differences in peak sensitivities (Table 1).

Ocular following responses are eye-tracking movements generated in response to sudden motion by large stimuli at very short latencies. In humans and macaques, the latency of these responses is reported to be <85 and <60 ms, respectively (Miles et al., 1986; Gellman et al., 1990; Busettini et al., 1994; Miles, 1998). We find that the marmosets also demonstrate short-latency tracking movements in response to large field motion with comparable latencies. Within the range of motion parameters we tested, we find that for single grating motions, the maximum response amplitude for marmoset ocular following responses is ∼1–2°/s. The response amplitude is stronger for stimuli composed of more complex patterns than gratings, such as a field of dots presenting multiple spatial frequencies. In this case, the maximum response amplitudes we observe are ∼3–5°/s. The lower amplitude with single gratings must therefore reflect poorer activation of involved neurons as the stimulus is restricted to a single frequency channel.

We find the marmoset and macaque ocular following spatial frequency selectivity to be similar when compared using gratings. Both species show higher gains at an SF of ∼0.3 cpd (macaque results from the study by Miles et al., 1986). Area MT, where neurons are selective for motion, is thought to contribute the major sensory drive for ocular following responses in primates, as evidenced by links between MT-MST neuronal responses and ocular following eye movements, the shorter latency of these neurons compared with OFR, and impaired OFR following lesions in these areas (Kawano et al., 1994; Takemura et al., 2007). Unlike the preferred spatial frequency of OFR, the preferred spatial frequency of motion-selective area MT neurons of marmosets is lower than that of the macaques [0.2 cpd in marmosets (Lui et al., 2007); 0.6 cpd in macaques (Priebe et al., 2003)]. One explanation for the higher SF preference in marmoset OFR could be the higher cone density in the marmoset peripheral retina compared with macaque and human retinas (Wilder et al., 1996; Mitchell and Leopold, 2015; Grünert and Martin, 2020).

The median optimal spatial frequency for area MT neuron responses in the marmoset is ∼0.2 cpd, and the median optimal temporal frequency is ∼3 Hz (Lui et al., 2007). This sensitivity matches the observed spatial frequency sensitivity of 0.2−0.4 cpd in the ocular following responses. Based on the MT neuron responses, the optimal speed should be ∼15°/s, and we observed there to be a response saturation at speeds >25°/s. Given the overlap in the peak sensitivity for these motion parameters between marmoset MT neuron responses and marmoset MT ocular following responses, it is possible for these MT responses to be underlying the ocular following responses in the marmoset. The small discrepancies in peak sensitivities between OFR and neural responses may be because of the contribution of MST neurons, which in macaques is known to have different sensitivity than MT (Miura et al., 2014). Finally, the gain in ocular following response to motion of different speeds, saturates earlier in the marmoset than the macaque. This may be a result of smaller head sizes and higher prevalence of head movements (not possible under head-fixed conditions) in marmosets when they are tracking motion (Pandey et al., 2020).

Human and macaque observers have been shown to successfully track the integrated trajectory of spatial patterns formed of multiple motion components (Masson and Castet, 2002; Barthélemy et al., 2008; Quaia et al., 2016). Marmosets also track such integrated motion when presented with plaid patterns. In the human and macaque studies, it was observed that the initial tracking component would be in response to the local motion component, whereas the response to the integrated motion has a slightly longer latency and tends to follow with a delay of 20 ms. In our records, the distinction between tracking directions in the early time intervals has been difficult to assess as the initial marmoset eye movements are small, causing the initial tracking direction estimates to be noisy.

The neural basis underlying the responses in the integrated motion direction is less clear. Marmoset MT, just like macaque MT, is composed of pattern cells, component cells, and cells in between the spectrum (Solomon et al., 2011). It remains unclear as to how this diverse MT population signals the visual motion direction as a whole (Quaia et al., 2022). Previous studies have demonstrated a contribution of multiple motion-sensing mechanisms, such as based on first-order motion, second-order motion, as well as features (Lorenceau et al., 1993; Masson et al., 2000; Barthélemy et al., 2008). Motion detection/integration can occur differently in each of these channels and together may contribute to the ocular following eye movements. It is also likely that selectivity for integrated global motion is enhanced in circuits following area MT, which finally contribute to guiding ocular following direction. This does not negate the possibility for subcortical contribution to ocular following responses (Inoue et al., 2000). The marmoset offers specific advantages for how we might untangle the motion signals for eye movements. Areas MT and MST, and the frontal eye movement regions are easily accessed for imaging or electrophysiology. The ability to train them to perform simple eye movement-dependent tasks and the similarity of visual pathway with macaques and humans, makes them well suited to study the neural basis of motion computations and explore how they guide behavior such as eye movements.

## Synthesis

Reviewing Editor: David Schoppik, New York University Decisions are customarily a result of the Reviewing Editor and the peer reviewers coming together and discussing their recommendations until a consensus is reached. When revisions are invited, a fact-based synthesis statement explaining their decision and outlining what is needed to prepare a revision will be listed below. The following reviewer(s) agreed to reveal their identity: NONE.

The reviewers were generally positive & felt that the study was a good fit for eNeuro. One suggestion to strengthen the manuscript was additional Discussion that made the case for the marmoset’s specific utility in studying area MT, given the abundance of prior work in the macaque.

Minor comments follow below.

Pg. 3- It would be useful to include citations for the sentence “Humans generally perceive...”

Top of page 4- Animals weighed 5-10% of baseline?

Pg. 4: What does “mean luminance 127” mean? (need units)

Pg. 4: What does it mean to fixate for 250-300ms “with a 220 ms grace period”? Why is that value not incorporated into the fixation duration?

Pg. 4: What does it mean that the stimulus “moved for 200-400 ms”? Did it have a random duration? Different stimuli had different durations?

Pg. 4: When was the reward delivered? What was the inter-trial interval?

Pg. 4: State if monitor is LCD or CRT technology.

Pg. 5: How many trials were collected for each condition (average, range)? What fraction of trials was excluded based on the drift and saccade criteria described?

Pg. 5: What does it mean that “the trace was differentiated at 50 and 25 Hz”?

Pg. 6: It is unclear how the OFR magnitude was computed. Here we are told that, if we indicate with t0 the latency (relative to stimulus onset) they subtracted from the average speed in the [2*t0-15ms, 2*t0] interval the average speed in the [t0-15ms, t0] interval. In page 8 (and in Figure 2) we are however told that they used the average speed in [t0+25ms, t0+40ms]. What was actually done? Note that in either case this would be a departure from typical OFR measurements, when typically what is measured is the average speed in the open loop period [t0, 2*t0], possibly subtracting the average speed in the [0, t0] period. Why such short windows are used here is not at all clear.

Pg. 7: If the fits “were calculated on a log scale” the equation should indicate it clearly, so that there is no ambiguity about what was done.

Pg. 8: (Top) Please you include what % of trials were discarded per animal?

Pg. 8: The “saccade initiated paradigm” is usually used because of the well-known post-saccadic enhancement of ocular following responses (Kawano & Miles, 1986. Short-latency ocular following responses of monkey. II. Dependence on a prior saccadic eye movement.)

Pg. 8: “comparable amplitude to those observes in the macaque”. Please provide reference(s). Also note that the overall amplitude of OFRs varies across subjects by a very large factor (at least 8), so it is not clear what value this information has, if any.

Pg. 8: (bottom) Animal A and B are now animal 1 and animal 2??

Pg. 9: What is the meaning of mean and standard deviation here (1st paragraph)? Does it refer to the fitting log-Gaussian? In that case the SD is meaningless as listed, since it would have to be in log units, not in deg/s. I’m not too enthusiastic about the fit for animal B (Fig. 3B), perhaps if the authors were to average speed over the entire open loop period it would be less noisy.

Pg. 9: The choice of measuring spatial frequency tuning by drifting sine gratings of various SFs at a constant speed is peculiar. It has been shown repeatedly that ocular following responses are (e.g., Miura, Inaba, Aoki and Kawano, J Neurosci, 2014; Sheliga, Quaia, FitzGibbon and Cumming, J Vis, 2016) or almost separable (Sheliga, Quaia, FitzGibbon and Cumming, J Vis, 2020) in the SF-TF plane. It is thus customary to measure SF tuning by using a constant temporal frequency (typically around 20Hz, the preferred TF for monkeys and humans). With the speed used here (25deg/s), the preferred TF is used for the highest SF stimulus used (0.8cpd), and lower SFs are handicapped by being tested at progressively less optimal TFs. The authors have thus inserted a positive bias in their SF tuning curves. This probably explains why they in humans and monkeys the preferred SF is usually found to be lower (around 0.25-0.3cpd) than what reported here. I realized that repeating the experiments in the appropriate manner would be a considerable burden, but this issue needs to at least be described and acknowledged. It should also be note that the preferred SF varies as a function of the size of the stimulus Sheliga, Quaia, FitzGibbon and Cumming, J Vis, 2012

Pg. 9: Again, the “standard deviation” of a log Gaussian curve cannot be expressed in this way; 3.9 means nothing, we are not even told which log base was used.

Pg. 10: Quaia, Optican and Cumming, J Neurosci, 2016 should be cited here, as it addresses and clarifies many issues that were left unresolved by Masson and Castet, 2002.

Pg. 11 “indicating a significant response...” The p-value for one animal is 0.1. Is that considered statistically significant?

Pg. 12 It would be useful at this point to have a table, in which the various tuning values extracted here for the marmoset are compared directly to those previously reported in humans and monkeys (with appropriate source citations). The authors talk about the similarities, but having a simple table would make the point in a more concise and convincing way.

Pg. 13- It would be useful to include mention of the latency of MT responses when discussing the possibility that they support OFR.

Pg. 14: Quaia, Optican and Cumming, J Neurosci, 2016 studied the nature of the 20ms delay, and reached conclusions that are somewhat different.

Last sentence of Discussion: typo “cortical and circuitry”. Also please change “ideal” to something else like “good” or “useful”

## References

[B1] Adelson EH, Movshon JA (1982) Phenomenal coherence of moving visual patterns. Nature 300:523–525. 10.1038/300523a0 7144903

[B2] Barthélemy FV, Perrinet LU, Castet E, Masson GS (2008) Dynamics of distributed 1D and 2D motion representations for short-latency ocular following. Vision Res 48:501–522. 10.1016/j.visres.2007.10.020 18221979

[B3] Batschelet E (1981) Circular statistics in biology. San Diego: Academic.

[B4] Busettini C, Miles FA, Schwarz U, Carl JR (1994) Human ocular responses to translation of the observer and of the scene: dependence on viewing distance. Exp Brain Res 79:484–494. 10.1007/BF00229187 7813684

[B5] Ferrera VP, Wilson HR (1990) Perceived direction of moving two-dimensional patterns. Vision Res 30:273–287. 10.1016/0042-6989(90)90043-K 2309462

[B6] Gellman RS, Carl JR, Miles FA (1990) Short latency ocular-following responses in man. Vis Neurosci 5:107–122. 10.1017/s0952523800000158 2278939

[B7] Grünert U, Martin PR (2020) Cell types and cell circuits in human and non-human primate retina. Prog Retin Eye Res 78:100844. 10.1016/j.preteyeres.2020.100844 32032773

[B8] Inoue Y, Takemura A, Kawano K, Mustari MJ (2000) Role of the pretectal nucleus of the optic tract in short-latency ocular following responses in monkeys. Exp Brain Res 131:269–281. 10.1007/s002219900310 10789943

[B9] Kawano K, Miles FA (1986) Short-latency ocular following responses of monkey. II. Dependence on a prior saccadic eye movement. J Neurophysiol 56:1355–1380. 10.1152/jn.1986.56.5.1355 3794773

[B10] Kawano K, Shidara M, Watanabe Y, Yamane S (1994) Neural activity in cortical area MST of alert monkey during ocular following responses. J Neurophysiol 71:2305–2324. 10.1152/jn.1994.71.6.2305 7931519

[B11] Lorenceau J, Shiffrar M, Wells N, Castet E (1993) Different motion sensitive units are involved in recovering the direction of moving lines. Vision Res 33:1207–1217. 10.1016/0042-6989(93)90209-f 8333170

[B12] Lui LL, Bourne JA, Rosa MGP (2007) Spatial and temporal frequency selectivity of neurons in the middle temporal visual area of new world monkeys (Callithrix jacchus). Eur J Neurosci 25:1780–1792. 10.1111/j.1460-9568.2007.05453.x 17432965

[B13] Masson GS, Castet E (2002) Parallel motion processing for the initiation of short-latency ocular following in humans. J Neurosci 22:5149–5163. 10.1523/JNEUROSCI.22-12-05149.2002 12077210 PMC6757741

[B14] Masson GS, Rybarczyk Y, Castet E, Mestre DR (2000) Temporal dynamics of motion integration for the initiation of tracking eye movements at ultra-short latencies. Vis Neurosci 17:753–767. 10.1017/s0952523800175091 11153655

[B15] Miles FA (1998) The neural processing of 3-D visual information: evidence from eye movements. Eur J Neurosci 10:811–822. 10.1046/j.1460-9568.1998.00112.x 9753150

[B16] Miles FA, Kawano K (1986) Short-latency ocular following responses of monkey. III. Plasticity. J Neurophysiol 56:1381–1396. 10.1152/jn.1986.56.5.1381 3794774

[B17] Miles FA, Kawano K, Optican LM (1986) Short-latency ocular following responses of monkey. I. Dependence on temporospatial properties of visual input. J Neurophysiol 56:1321–1354. 10.1152/jn.1986.56.5.1321 3794772

[B18] Mitchell JF, Leopold DA (2015) The marmoset monkey as a model for visual neuroscience. Neurosci Res 93:20–46. 10.1016/j.neures.2015.01.008 25683292 PMC4408257

[B19] Mitchell JF, Priebe NJ, Miller CT (2015) Motion dependence of smooth pursuit eye movements in the marmoset. J Neurophysiol 113:3954–3960. 10.1152/jn.00197.2015 25867740 PMC4485767

[B20] Miura K, Inaba N, Aoki Y, Kawano K (2014) Difference in visual motion representation between cortical areas MT and MST during ocular following responses. J Neurosci 34:2160–2168. 10.1523/JNEUROSCI.3797-13.2014 24501357 PMC6608534

[B21] Pandey S, Simhadri S, Zhou Y (2020) Rapid head movements in common marmoset monkeys. IScience 23:100837. 10.1016/j.isci.2020.100837 32058952 PMC6997856

[B22] Pattadkal JJ, Zemelman BV, Fiete I, Priebe NJ (2022) Primate neocortex performs balanced sensory amplification. BioRxiv 497220. 10.1101/2022.06.23.497220

[B23] Priebe NJ, Cassanello CR, Lisberger SG (2003) The neural representation of speed in macaque area MT/V5. J Neurosci 23:5650–5661. 10.1523/JNEUROSCI.23-13-05650.2003 12843268 PMC2553808

[B24] Quaia C, Optican LM, Cumming BG (2016) A motion-from-form mechanism contributes to extracting pattern motion from plaids. J Neurosci 36:3903–3918. 10.1523/JNEUROSCI.3398-15.2016 27053199 PMC4821905

[B25] Quaia C, Kang I, Cumming BG (2022) Pattern motion direction is encoded in the population activity of macaque area MT. J Neurosci 42:9372–9386. 10.1523/JNEUROSCI.0011-22.2022 36332976 PMC9794370

[B26] Sheliga BM, Quaia C, Fitzgibbon EJ, Cumming BG (2016) Ocular-following responses to white noise stimuli in humans reveal a novel nonlinearity that results from temporal sampling. J Vis 16(1):8, 1–22. 10.1167/16.1.8 26762277 PMC4743714

[B27] Solomon SS, Tailby C, Gharaei S, Camp AJ, Bourne JA, Solomon SG (2011) Visual motion integration by neurons in the middle temporal area of a New World monkey, the marmoset. J Physiol 589:5741–5758. 10.1113/jphysiol.2011.213520 21946851 PMC3249047

[B28] Takemura A, Murata Y, Kawano K, Miles FA (2007) Deficits in short-latency tracking eye movements after chemical lesions in monkey cortical areas MT and MST. J Neurosci 27:529–541. 10.1523/JNEUROSCI.3455-06.2007 17234585 PMC2430053

[B29] Wilder HD, Grunert U, Lee BB, Martin PR (1996) Topography of ganglion cells and photoreceptors in the retina of a New World monkey: the marmoset *Callithrix jacchus*. Vis Neurosci 13:335–352. 10.1017/S0952523800007586 8737285

[B30] Yo C, Wilson HR (1992) Perceived direction of moving two-dimensional patterns depends on duration, contrast and eccentricity. Vision Res 32:135–147. 10.1016/0042-6989(92)90121-x 1502799

